# Patient-reported long-term benefit with an active transcutaneous bone-conduction device

**DOI:** 10.1371/journal.pone.0241247

**Published:** 2020-11-02

**Authors:** Julia Hundertpfund, Jens Eduard Meyer, Attila Óvári

**Affiliations:** 1 Asklepios Medical School, Semmelweis University, Hamburg, Germany; 2 Department of Oto-Rhino-Laryngology, Head and Neck Surgery, Plastic Surgery, Asklepios Klinik St. Georg, Hamburg, Germany; 3 Department of Oto-Rhino-Laryngology, Head and Neck Surgery “Otto Koerner”, University Medical Center, Rostock, Germany; Universidade Federal de Sao Paulo/Escola Paulista de Medicina (Unifesp/epm), BRAZIL

## Abstract

**Purpose:**

To evaluate the long-term benefits in hearing-related quality of life, patient satisfaction and wearing time of patients rehabilitated with an active transcutaneous bone-conduction device. Adverse events and audiological outcomes are reported as secondary outcomes.

**Methods:**

This retrospective, mono-centric cohort analysis involves 16 adults with conductive or mixed hearing loss with a mean device experience of 51.25 months. Patient-reported outcome measures were assessed using the short version of the Speech, Spatial and Qualities of Hearing Scale (SSQ12-B) and the German version of the Audio Processor Satisfaction Questionnaire (APSQ). Audiological outcomes as well as incidence of adverse events were obtained from patients´ charts.

**Results:**

The hearing-related quality of life improved significantly within all subscales of the SSQ12-B scoring a mean overall of 2.95 points. Patient satisfaction measured with the APSQ scored 8.8 points on average. Wearing times differed considerably and patients with lower levels of education seemed to use their device longer compared to patients with academic education. Eight minor adverse events were documented, all of which resolved during follow-up. The mean gain in word recognition score at the last follow-up measured at 65 dB was 75.9%, while speech reception threshold was lowered by 35.1 dB.

**Conclusion:**

Even after several years, patients report significant benefits in hearing-related quality of life and device satisfaction. In combination with a low rate of minor adverse events and significantly improved audiological outcomes, the device is considered as a comfortable and effective option in hearing rehabilitation.

## Introduction

Any kind of hearing loss leads to deficits in social, emotional and educational development [1]. One of the main goals of the treatment is the successful social reintegration in professional or private life. Therefore, patient satisfaction and quality of life (QoL) in hearing rehabilitation are as important as beneficial audiologic outcome. The evaluation of patient-reported outcome measures (PROMs) enables to place the patient at the center of his treatment by measuring subjective benefits and experiences [2]. Bone-conduction devices (BCDs) can be used to restore conductive (CHL) and mixed hearing loss (MHL) [3]. Thereby malfunction of the external and middle ear can be circumvented by transmitting vibrations that are produced by the device through the cranial bone to the inner ear [3, 4]. Since active transcutaneous BCDs do not rely on osseointegration and do not require constant penetration of the skin, the occurrence of an adverse event is less frequently than with percutaneous implants, but with comparable beneficial audiological outcome [5]. Hypothetically, transcutaneous BCDs may lead to improved QoL, especially because of the advantages of the intact skin with less discomfort, no requirement of regular hygienic maintenance and no social stigma [6–8]. The aim of this study was to retrospectively evaluate the long-term benefits in hearing related QoL, patient satisfaction and wearing time of an active transcutaneous BCD. Adverse events and audiological outcomes are reported as secondary outcomes.

## Materials and methods

### Study design and ethical approval

The relevant clinical parameters were taken from the outpatients and inpatients medical records. These included the evaluation of audiometric data, surgical and medical reports as well as the QoL-questionnaires.

The study protocol was approved by the ethics committee at the University of Luebeck (EC.No. 17-164A). The principal investigator was JEM. Because of the retrospective design, a further informed consent was not necessary as all examinations and the distribution of questionnaires were carried out as part of clinical routine and on a voluntary basis. Any identifying information (name, date of birth) was removed from the data set before analysis.

### Study population

In total, 16 adult patients with CHL or MHL were included. The study population received unilateral implantation of the bone-conduction hearing device Bonebridge™ BCI-601 (MED-EL, Innsbruck, Austria) between September 2012 and December 2018 at the ENT department of the Asklepios Clinic St. Georg, Hamburg, Germany.

### Patient-reported outcome measures (PROMs)

PROMs were collected using the German versions of the Speech, Spatial and Qualities of Hearing Scale (SSQ12-B) and the Audio Processor Satisfaction Questionnaire (APSQ) questionnaires.

The SSQ12-B was used to measure patient-reported hearing benefits in a variety of everyday listening situations. Patients were asked to compare their hearing abilities in the aided condition versus the situation before the implantation on a Likert-scale ranging from -5 (much worse) to +5 (much better) and 0 indicating no difference [9]. The SSQ-B(enefit), unlike other versions that evaluate subjective satisfaction at the time of the survey, focuses on the subjective benefit compared to the state before implantation.

The APSQ contains 15 items and three subscale scores (wearing comfort, social life, usability) with 5 items each. Patients were asked to rate their satisfaction with the audio processor in described everyday situations on a visual analog scale (VAS) from 0 (do not agree at all) to 10 (agree completely) [10].

The wearing time was evaluated in hours per day and night. Patients were divided into different groups: firstly, into patients with higher and lower level of education, which is defined by not having obtained a high school degree. Secondly, in patient’s employment situation, whereby retirees were included in the group of unemployed patients. Thirdly, into four different age groups (0–20, 21–40, 41–60 and 61–80 years).

### Audiometric testing

For all audiometric tests the audiometer Auritec AT900 (Hamburg, Germany) was used and the tests took place in a soundproof audiometric booth (Industrial Acoustics Company, Winchester, United Kingdom).

Pure tone measurements were performed at a frequency range from 0.25 kHz to 8 kHz. Pure tone average air (PTA_4AC_) and bone (PTA_4BC_) conduction hearing thresholds were calculated as the mean of the evaluated AC and BC values at 0.5, 1, 2 and 4 kHz.

Sound field thresholds were measured using continuous warble tones presented from the aided side (S90), with the loudspeaker positioned 1 m away from the subject. The contralateral ear was masked with narrow-band noise through headphones.

The word recognition score (WRS) and speech recognition thresholds (SRT50) were assessed with the German Freiburger monosyllables test. The words were presented at 65 dB and 80 dB SPL. Both, WRS and SRT50 were measured in aided (sound field) and unaided (headphones) condition, whereas the non tested ear was masked with broadband noise through headphones.

Single values that could not be tested due to the audiometer threshold being reached were replaced by the maximal audiometer output limit (110 dB) for AC and speech reception testing.

### Data analysis

All relevant data were extracted from patient charts into excel tables and can be found as S1 Dataset. Descriptive statistics were used to report demographics (e.g. age and gender), baseline characteristics (e.g. etiologies), patient-reported outcomes and wearing time. The non-parametric Wilcoxon signed-rank test was used to test for significant differences between unaided and aided pure tone, free-field, WRS and SRT50 outcomes. Scores from the SSQ12-B questionnaire were analysed using the one-sample Wilcoxon signed-rank test to test for significant difference of the median score to zero (0 = no change in QoL).

The α-level was set to p<0.05. Statistical tests of patient reported outcomes were performed with R v3.4.1 via the RStudio v1.0.126 [11] integrated development environment. GraphPad Prism 6.0 was used for the statistical analysis of audiological outcomes and the preparation of all graphs.

## Results

### Patient demographics

The mean age at the time of implantation of the nine men and seven women was 45.06 years (SD ±17.47). At the time of last follow-up the average experience with the device was 51.25 months (±21.58), with a maximum of 84 months and a minimum of 6 months after implantation. Detailed patient demographics and medical histories are summerized in Table 1.

**Table 1:** Demographics.

Patient-ID	Age at the time of implantation, years	Gender	Disease etiology	Implant side	HL- type	Localization of the FMT	Surgery performed in parallel to BCI implantation	Previous surgeries (implanted side only)	Follow-up time, months
Pat. 1	50	M	Cholesteatoma, ossicular chain disruption	L	CHL	TM	X	2x tympanoplasties, implantation of an alloplastic prosthesis, radical mastoidectomy,	72
Pat. 2	27	M	Atresia auris, ear canal stenosis	L	CHL, bilateral	TM	X	Reconstruction of the ear canal	47
Pat. 3	64	F	Tympanosclerosis	L	MHL, bilateral	TM	X	Multiple tympanoplasties, implantation of an alloplastic prosthesis, middle ear surgery	75
Pat. 4	52	M	Post-inflammatory meatal fibrosis	L	CHL	TM	X	Tympanoplasty, mastoidectomy, surgery and revision surgery of the ear canal	76
Pat. 5	16	M	Atresia auris, microtia	R	MHL	TM	Reconstruction of the pinna stage 2	Reconstruction of the pinna stage 1	54
Pat. 6	62	M	Otitis externa, CSF- fistula, meningocele	R	CHL	slightly higher than usual (3-4mm above the cranial calotte)	Petrosectomy, mastoid revision surgery, obliteration of the middle ear with abdominal fat, duraplasty, resection of a meningocele and closure of the skull	Multiple tympanoplasties, radical mastoidectomy, closure of a CSF-fistula	36
Pat. 7	30	F	Otosclerosis	L	MHL, CHL contralateral	RS	Dilation of the Eustachian tube, ear microscopy	Multiple tympanoplasties, radical mastoidectomy, dilation of the Eustachian tube	60
Pat. 8	50	F	Ossicular chain disruption	L	CHL, bilateral	TM	Tympanotomy and removal of dislocated prosthesis	Tympanoplasty, implantation of an alloplastic prosthesis, explorative tympanotomy, middle ear surgery	45
Pat. 9	51	M	Microtia, malformation of the middle ear	L	MHL, bilateral	TM	X	Multiple tympanoplasties, implantation of an alloplastic prosthesis	70
Pat. 10	53	M	Cholesteatoma, conventional hearing aids not tolerated	R	MHL, bilateral	RS	Tympanotomy and removal of dislocated prosthesis	Multiple tympanoplasties, implantation of an alloplastic prosthesis, radical mastoidectomy	84
Pat. 11	36	F	Chronic otitis media otosclerosis, tympanosclerosis	R	CHL	TM	Duraplasty	Tympanoplasty, implantation of an alloplastic prosthesis, atticotomy	46
Pat. 12	14	M	Cholesteatoma	L	MHL	RS	X	Tympanoplasty, radical mastoidectomy, revision surgery of the radical cavity	58
Pat. 13	75	M	Cholesteatoma, surgical obliteration of the ear canal	L	MHL, CHL contralateral	TM	Mastoidectomy	Partial petrosectomy	21
Pat. 14	55	F	Chronic otitis media	L	CHL	RS	Mastoidectomy, petrosectomy, obliteration of the middle ear with abdominal fat, duraplasty, closure of the Eustachian tube	Tympanoplasty, radical mastoidectomy, revision surgery of the radical cavity, tympanoscopy, tympanostomy tube, dilation of the Eustachian tube	32
Pat. 15	55	F	Chronic otitis media, ossicular chain disruption, labyrinthitis, conventional hearing aids not tolerated	R	CHL	TM	X	Tympanoplasty, radical mastoidectomy, revision surgery of the radical cavity, tympanostomy tube, atticotomy	38
Pat. 16	31	F	Atresia auris, malformation of the middle ear, ear canal stenosis, ear dysplasia	L	CHL	TM	X	Tympanoplasty, implantation of an alloplastic prosthesis, reconstruction of the ear canal, eardrum reconstruction	6

F, female; M, male; L, left; R, right; CHL, conductive hearing loss; MHL, mixed hearing loss; TM, transmastoidal; RS, retrosigmoidal; X, no additional surgery

### Patient- reported outcome measures

#### SSQ12-B

**Fig 1:**
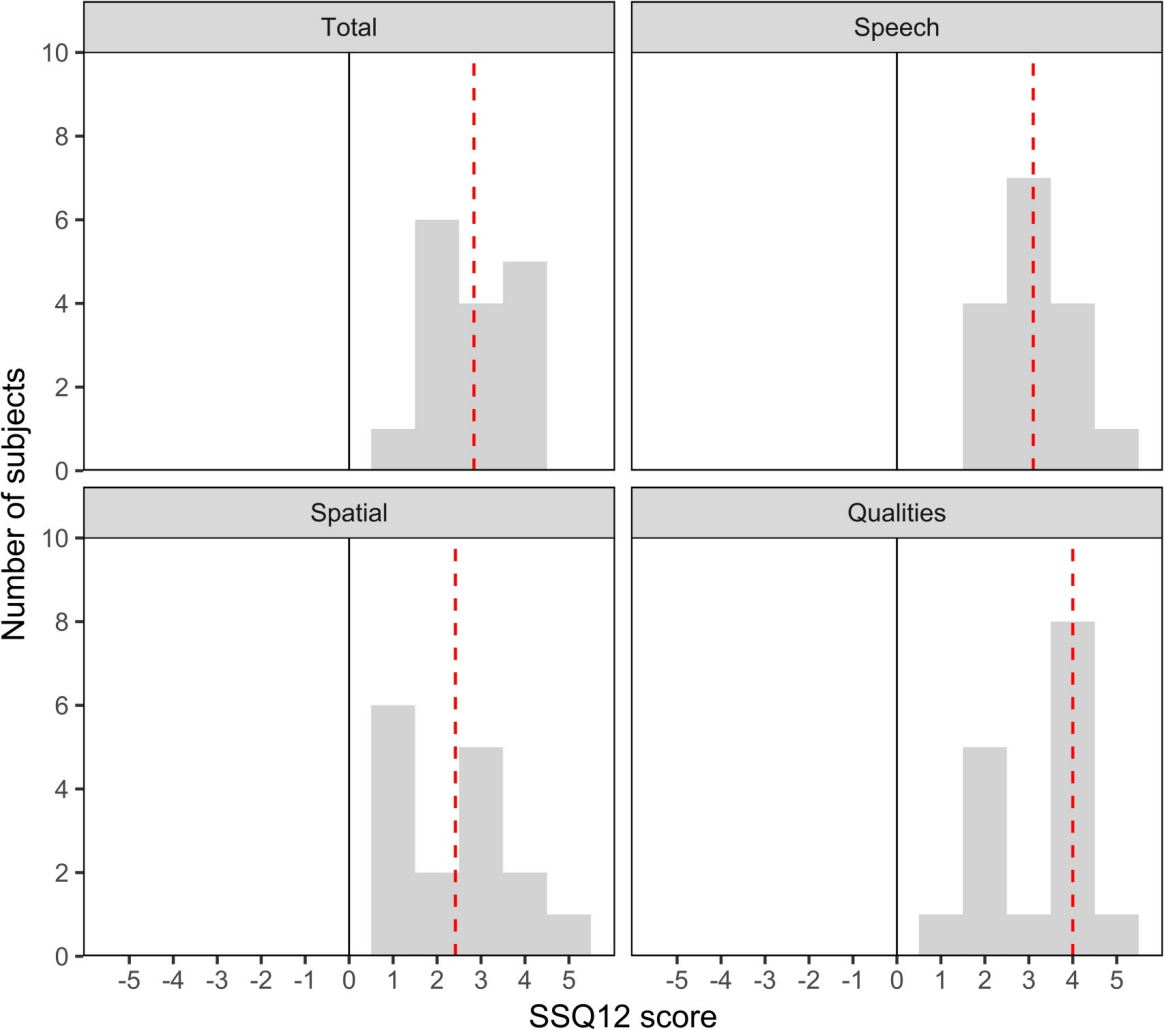
SSQ12-B. The SSQ12-B diagram shows the frequency of subjective scores ranging from -5 to +5 in every subcategory (“spatial”, “speech”, “qualities”) and in the overall score (“total”). The mean is given as dotted line.

The overall SSQ12-B score revealed a significant subjective benefit (Wilcoxon signed-rank test, p<0.001) in patients speech intelligibility and sound localization, as well as the quality of hearing, with a mean overall score of 2.95 points (SD ±0.942). In all three subcategories significant improvement was reported. The mean SSQ subcategory “speech” was scored 3.06 (SD ±0.991, Wilcoxon signed-rank test, p<0.001), “spatial hearing” was scored 2.42 (SD ±1.23, Wilcoxon signed-rank test, p = 0.0004) and the “quality of hearing” 3.25 (SD ±1.21, Wilcoxon signed-rank test, p = 0.0004) (Fig 1).

Item-wise mean scores are summerized in Table 2.

**Table 2:** Item-wise analysis of the SSQ12-B and APSQ.

Item-wise analysis	SSQ12-B			APSQ		
Item	mean	median	+/- SD	mean	median	+/- SD
**Item 1**	4.00	4.00	0.75	8.69	9.75	2.57
**Item 2**	2.72	3.00	2.18	9.78	10.00	0.31
**Item 3**	2.56	3.00	1.82	9.06	10.00	1.86
**Item 4**	2.84	3.00	1.56	8.23	9.50	2.65
**Item 5**	3.16	3.00	1.18	9.25	10.00	1.98
**Item 6**	2.09	1.75	1.73	9.22	9.50	1.02
**Item 7**	2.06	2.00	1.31	9.16	10.00	1.42
**Item 8**	3.09	3.50	1.45	8.41	9.75	2.71
**Item 9**	2.53	3.25	1.90	9.39	9.50	0.88
**Item 10**	2.93	4.00	2.15	8.,50	9.50	2.34
**Item 11**	3.88	4.00	0.94	9.44	10.00	1.34
**Item 12**	3.59	4.00	1.34	4.37	3.00	4.11
**Item 13**				9.34	9.75	1.08
**Item 14**				9.44	10.00	0.93
**Item 15**				9.63	10.00	0.99

SSQ12-B, Speech, Spatial and Qualities of Hearing Scale; APSQ, Audio Processor Satisfaction Questionnaire

#### APSQ

**Fig 2:**
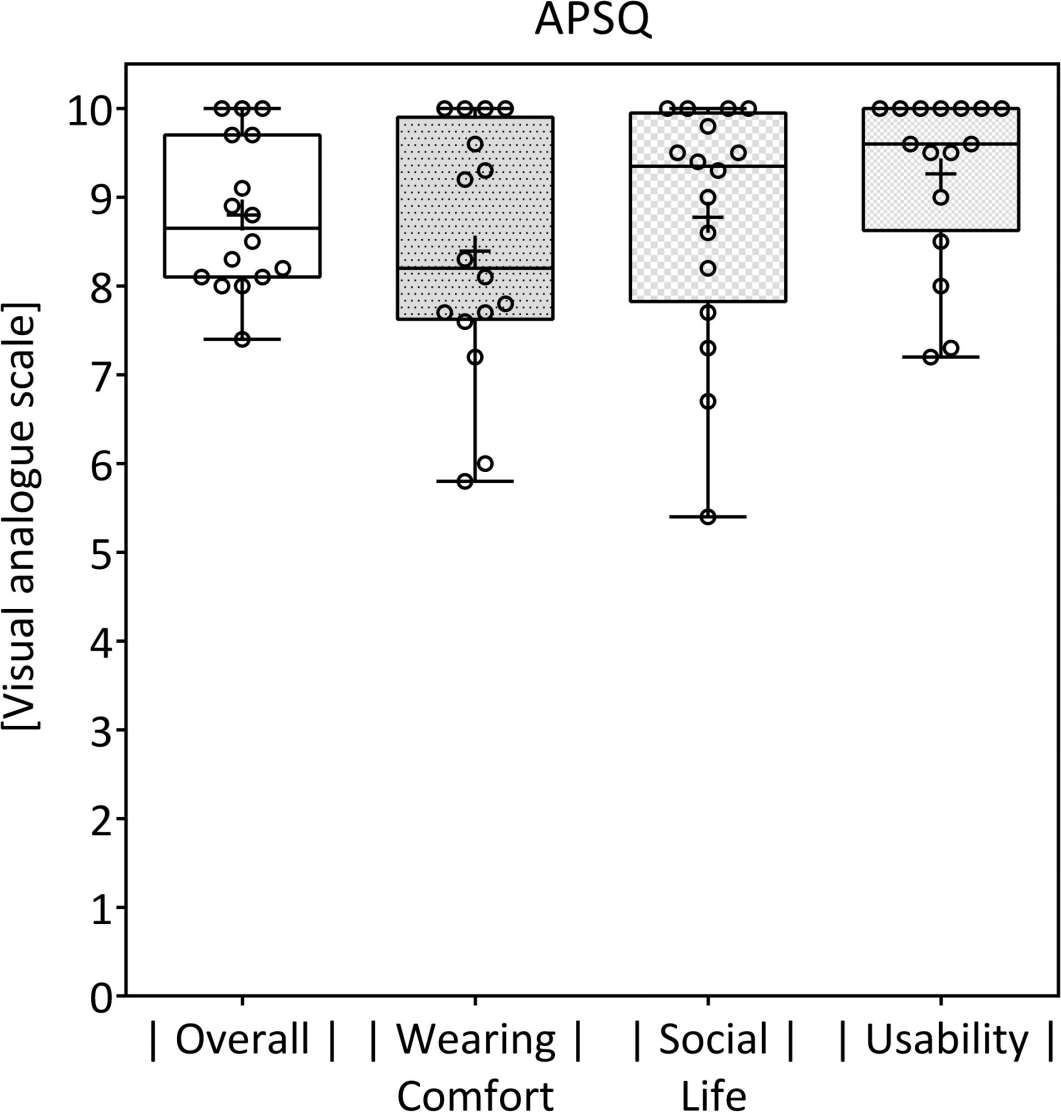
APSQ. Results of the APSQ (“overall”) and its subscores (“wearing comfort", “social life”, “usability”). Boxes indicate the 25^th^ and 75^th^ percentiles. Mean and median are given as plus signs (+) and horizontal lines. Minimum and maximum values are depicted by whiskers. The points represent the individual results of every patient. The item-wise analysis can be found in Table 2.

The mean overall APSQ scored 8.8 (SD ±0.854). The mean “social life” subscore was 8.77 (SD ±1.38). The mean “usability” component scored 9.26 (SD ±0.983) and the mean “wearing comfort” 8.39 (SD ±1.4) (Fig 2).

#### Wearing time

During the first audio processor adjustment, the audio processor is individually adapted to each patient, whereby in most cases the magnet strength of 2–3 (out of 5) is usually selected at the beginning. During follow-up this strength is modulated in the best possible way to provide maximum comfort and hearing benefit.

Seven patients reported to wear their audio processor more than 12 hours per day, four used it for 9–12 hours, two for 6–9 hours, one for 3–6 hours and one for 1–3 hours. Most patients did not wear their audio processor at night (n = 14), while one patient wore it approximately in 50% of the nights to be able to hear her newborn and another patient wore it always at night (100%) due to personal preference.

**Fig 3:**
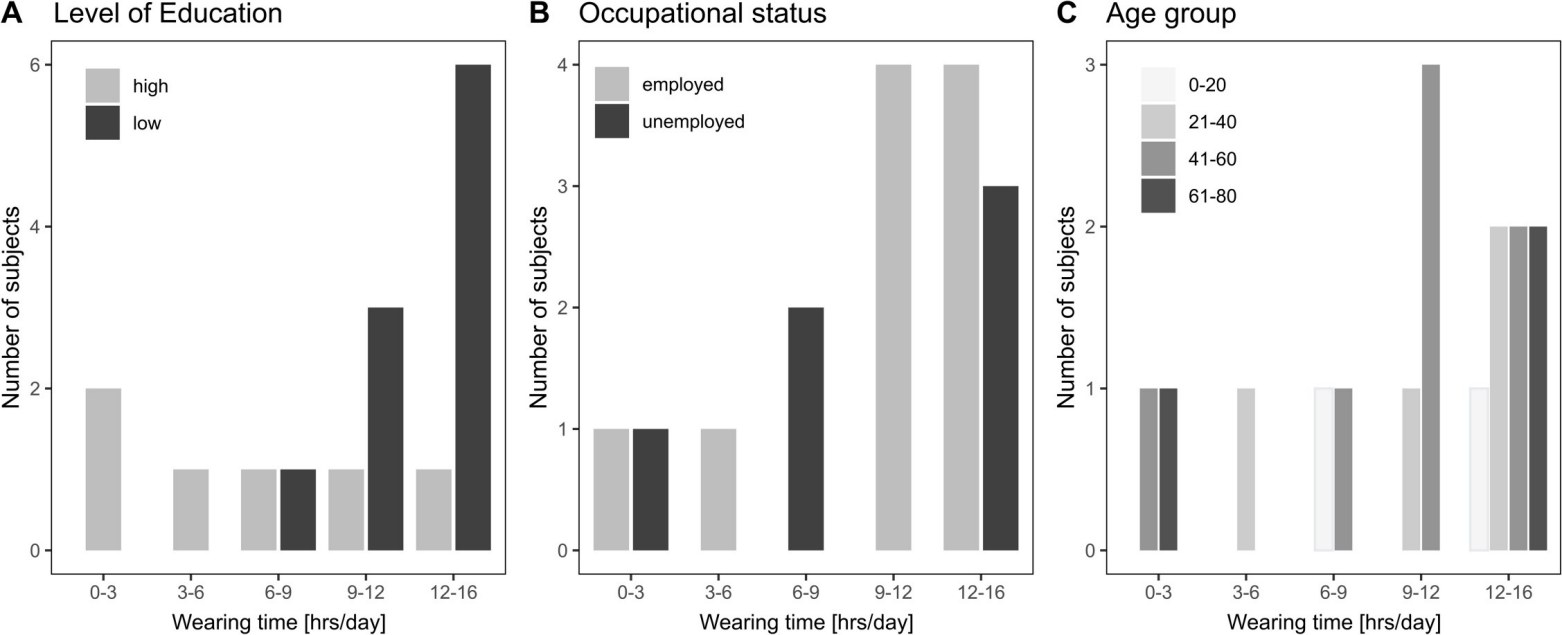
Wearing time. Distribution of wearing time categories as reported by 16 patients, plotted separately by level of education (A), occupational status (B) and age group (C).

The wearing time was higher in patients with lower level of education (Fig 3A). On the other hand, the wearing time was relatively independent from patient’s employment situation (Fig 3B), and age did not seem to influence wearing times (Fig 3C).

### Surgical outcomes and adverse events

To plan optimal placement of the bone conduction floating mass transducer (BC-FMT), CT scans of the temporal bone were performed preoperatively. In eleven patients the transmastoidal (TM) approach and in four patients the retrosigmoidal (RS) approach were carried out. Eight out of 16 patients underwent further surgical procedures additional to BCD implantation within the same setting (Table 1).

No complications occurred during surgery. BCI lifts were used in five patients (4 mm lifts in patients 6 and 14, 3 mm lifts in patients 8 and 15, and 1 mm lifts in Pat. 12). In case of Pat. 5, the rescue screw had to be used. Bone covering the dura and sigmoid sinus had to be partially removed in 6 cases (patients 1, 3, 6, 7, 9 and 12). The sigmoid sinus had to be compressed 3–4 mm in patients 9 and 12. In Pat. 12 the dura had to be compressed by 1 mm.

In the immediate postoperative period, defined as the time from surgery until activation of the implant (in general 4–6 weeks after surgery), six minor adverse events were observed and all were resolved until activation: PTA_4BC_ threshold dropped ≥ 10 dB in one patient (Pat. 6). BC-thresholds normalized during follow-up. Pat. 3 reported positional vertigo without nystagmus and problems associated with the ear pressure equalization 22 days after surgery. A small superficial wound dehiscence was observed in Pat. 5 on the lower line of incision. One case of atrophic skin (Pat. 13), one case of postoperative swelling (Pat. 11) and one case of a slightly painful haematoma (Pat. 14) in the implant area were noted, while the wound healing process was normal in these cases. Pat. 6 suffered from skin irritation and necrosis of the skin at the edge of the scar. All wound healing problems resolved until the activation of the implant.

During the follow-up time two minor adverse events were observed: Pat. 7 reported painful sensations in the implant area on very cold days without having any signs of skin irritation, lasting for six months postoperatively. Approximately two years postoperatively Pat. 14 developed a painful ulcer in the implant area. Consequent wound management and reduced magnet strength (1 out of 5) led to a complete healing.

### Pure tone and speech audiometry

#### Pure tone audiometry and sound field thresholds

**Fig 4:**
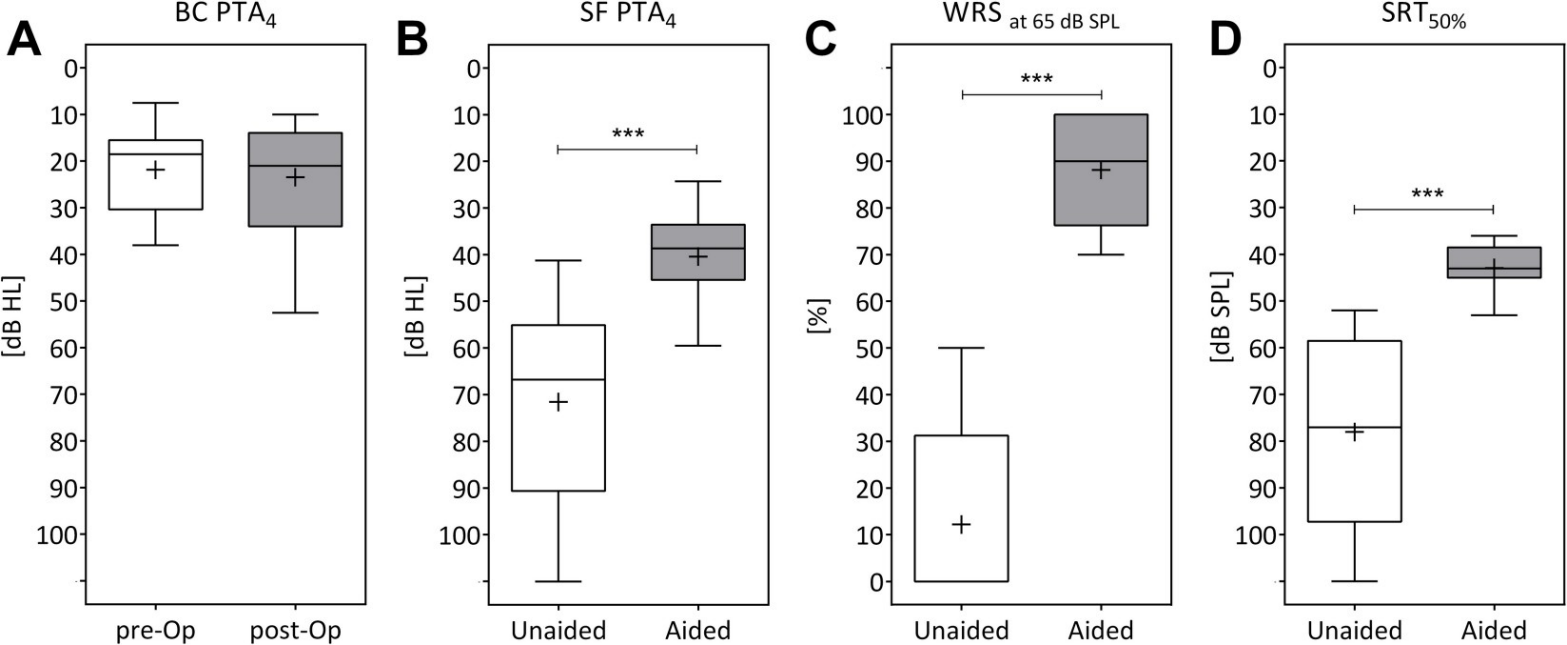
Audiological outcomes. Audiologic results comparing pre- with postoperative outcomes of bone conduction thresholds (4A), aided and unaided outcomes of hearing thresholds in sound field (4B), word recognition score at 65 dB SPL (4C) and 50% speech reception thresholds (4D). Boxes indicate the 25^th^ and 75^th^ percentiles. Mean and median are given as plus signs (+) and horizontal lines. Minimum and maximum values are depicted by whiskers; *** p<0.001.

Postoperative BC thresholds, measured at the last follow-up examination, were compared to preoperative measurements in order to assess the stability of the PTA_4BC_ and therefore the safety of the active transcutaneous BCD. The mean preoperative PTA_4BC_ was 21.8 dB HL (SD ±9.7), which was not significantly different (Wilcoxon signed-rank test, p = 0.2889) from the mean PTA_4BC_ of 23.4 dB HL (SD ±12.0) after implantation (Fig 4A).

The mean aided sound field threshold, PTA_4BB_ was 40.4 dB HL (SD ± 9.6). This implied that a significant functional gain (FG) of 31.2 dB (SD ±15.0) was achieved, compared to the mean unaided threshold PTA_4AC_ 71.6 dB HL (SD ±21.1, Wilcoxon signed-rank test, p<0.001) (Fig 4B).

#### Word Recognition Score (WRS) and Speech Reception Thresholds (SRT)

Speech intelligibility (WRS) in quiet measured at 65 dB showed a significant mean speech gain of 75.9% (SD ±13.9, Wilcoxon signed-rank test, p<0.0015). The mean unaided WRS at 65 dB was 12.2% (SD ±18.4) and in the aided condition the WRS at 65 dB was 88.1% (SD ±11.7) (Fig 4C). The WRS at 80 dB had also improved significantly by 59.1% (SD ±38.3), comparing the mean unaided WRS at 80 dB (39.7%, SD ±39.4) to the mean aided WRS (98.8%, SD ±3.9). The median in unaided condition was 30% (min: 0, max: 100) and in aided condition 100% (min 85, max 100), indicating a ceiling effect at 80 dB SPL.

The mean SRT50 improvement with the BCI was 35.1 dB (SD ±19.5). In comparison with the mean unaided SRT50, which was 78.0 dB (SD ±20.8), the mean aided SRT50 was 42.9 dB (SD ±4.9) (Wilcoxon signed-rank test, p<0.001) (Fig 4D).

Detailed audiometric outcomes are summerized in Table 3.

**Table 3:** Audiometric data.

Patient-ID	PTA_4BC_ pre-op.	PTA_4BC_ post-op directly after surgery	PTA_4BC_ post-op at the last follow-up	PTA_4AC_ unaided	PTA_4AC_ aided	FG	WRS65 unaided	WRS65 aided	Speech Gain	WRS80 unaided	WRS80 aided	WRS80 Improvement	SRT50 unaided	SRT50 aided	SRT50 Improvement
**Pat. 1**	16.25	14.75	12.25	45.25	30.00	15.25	45	100	55	80	100	20	53	42	11
**Pat. 2**	15.50	7.00	13.50	41.25	32.50	8.75	35	100	65	95	100	5	57	43	14
**Pat. 3**	38.00	43.00	37.50	63.25	42.25	21.00	0	70	70	30	100	70	73	45	28
**Pat. 4**	10.50	15.25	16.00	54.25	39.25	15.00	50	100	50	80	100	20	52	43	9
**Pat. 5**	28.50	26.75	24.25	79.00	37.50	41.50	0	95	95	30	100	70	95	40	55
**Pat. 6**	15.50	31.75	10.75	66.75	38.00	28.75	0	85	85	5	95	90	81	43	38
**Pat. 7**	21.00	20.25	23.25	66.75	41.00	25.75	0	90	90	80	100	20	65	42	23
**Pat. 8**	19.00	17.00	17.25	66.25	43.50	22.75	0	90	90	5	100	95	86	48	38
**Pat. 9**	34.50	44.00	37.00	97.00	46.00	51.00	0	75	75	0	100	100	110	53	57
**Pat. 10**	38.00	26.00	52.50	107.0	59.50	47.50	0	70	70	0	85	85	103	37	66
**Pat. 11**	17.00	17.50	18.75	50.75	35.00	15.75	10	80	70	65	100	35	60	38	22
**Pat. 12**	31.00	29.75	36.25	94.50	54.75	39.75	0	90	90	0	100	100	98	43	55
**Pat. 13**	27.00	24.25	27.25	110.0	54.50	55.50	0	70	70	0	100	100	110	51	59
**Pat. 14**	7.50	n.a.	15.25	70.00	33.25	36.75	0	95	95	0	100	100	87	37	50
**Pat. 15**	18.00	19.75	23.25	57.50	34.50	23.00	35	100	65	65	100	35	58	45	13
**Pat. 16**	12.25	10.25	10.00	75.00	24.25	50.75	20	100	80	100	100	0	60	36	24

op, operatively; n.a., not available; PTA4: pure tone average of the hearing thresholds measured at 0,5, 1, 2 and 4 kHz; AC, air conduction; BC, bone conduction; FG, functional gain; WRS, word recognition score; SRT, speech reception threshold.

## Discussion

The primary goal of this study was to evaluate patient-reported benefit and device satisfaction with an active transcutaneous BCD after several years of experience. We analysed data from the SSQ12-B questionnaire reveiling an improved hearing-related QoL after implantation in patients with CHL or MHL.

To our knowledge, only two comparable studies reported results on the SSQ questionnaires in patients treatet with this BCD: Laske et al. used the SSQ49-B (scale from -5 to +5) and Eberhard et al. used the SSQ12-A (scale from 1 to 10) [12, 13]. Laske et al. reported that for a patient cohort with single-sided-deafness (SSD), the “speech”component scored highest, while there was little improvement (close to 0) in the “spatial hearing”and “hearing qualities”subcategories [12]. However, Eberhard et al. surveyed patients with CHL, MHL and SSD and indicated improvement in the components “hearing qualities”and “spatial hearing”[13]. In our study consisting of patients with CHL and MHL, improvement was demonstrated in all subcategories, while the “hearing qualities” component was rated highest. The differences in the results of the SSQ-subscores may partially rely on the hearing capability of the contralateral ear.

The APSQ reported very high patient satisfaction with the audio processor and showed a tendency towards a ceiling effect, which was already described by Billinger-Finke et al. [10]. The authors reported comparable positive results of hearing device users (n = 69), who rated the “wearing comfort”noticeably low due to feedback problems when wearing hats, while all other items scored much higher.

Two patients (Pat. 3 and 10) were implanted at the “practical” indication limit, but their subjective benefit was different, which may indicate that indication limit and audiological/subjective benefit are not necessarily related. The subjective benefit of Pat. 10 was lower compared to other patients. With an APSQ overall score of 7.43 (out of maximal 10) points he seemed to have the lowest benefit of all patients and also scored a relatively low benefit on SSQ12 with an overall score of 1.96 points (from -5: much worse to +5: much better). On the other hand, Pat. 3, who was also implanted at indication limit, scored well in both questionnaires with an APSQ overall score of 9.73 points and an SSQ12-score of 4.5 points. Both patients were least satisfied in the sub-categories wearing comfort (APSQ) and spatial hearing (SSQ12).

The wearing time in hours per day indicates the effectiveness of the device, which was already reported in five other studies showing an average daily use of more than eight hours per day in children and adults [14–18], supporting the results of this study. Yang et al. reviewed the wearing time of 15 BCD-users and found that the wearing time was higher in patients with a higher level of education [18]. Interestingly, the wearing time in our study was higher in patients with lower level of education. It has been described that children with cochlear implants and a mainstream educational placement do also wear their device longer (at least 8h per day), and additional factors such as younger age at the time of implantation, oral mode of communication, higher maternal educational status affected the wearing time positively as well [19, 20]. Korkmaz et al. reported that the satisfaction of adult hearing aid users was lower with a lower level of education and shorter daily use [21], while Winn did not find a significant correlation between wearing time and employment situation [22]. Some of the higher educated patients in this study indicated that they prefer working in a quiet environment and therefore usually do not use their device at work. One patient (Pat. 14), who also wore the audio processor at night, developed a painful ulcer during follow-up. The magnet stregth was previously set at 3 (out of 5) and had already been reduced to 2, as the skin area showed a slight reddening most likely due to the long wearing time. Despite this, the patient developed an ulcer, but consistent wound management and the reduction of the magnet streght to 1 led to a complete healing. In the literature no comparable results concerning the magnet strength could be found, which might be important for better adjustment in the future. In most comparable studies no intra- or postoperative complications or adverse events of patients inside indication range had been published [4, 18, 23–28]. Some authors reported minor adverse events during follow-up like vertigo, tinnitus, pain, haematoma, wound healing disorder, swelling and skin irritation and/or infection [12, 13, 29–33]. No major adverse events, defined by the need for revision surgery or explantation, occurred in our study. In the literature only ten major adverse events were described so far: one revision surgery due to headache [31], one implant removal because of local infection [34], one explantation due to device failure [33], one case of sudden loss of hearing benefit [31], two explantations due to lack of benefit, while these patients were outside of indication criteria [25, 35] and four explantations due to wound dehiscence, mainly (3 cases) after implantation in a radical cavity [35]. According to other studies [12, 23, 26, 32], our experience has also shown that neither the RS-approach, which is the prefered device localization in patients with radical cavities, nor the sinus and/or dura exposition and compression, or the usage of BCI lifts had any negative influence on patients outcome. Furthermore, no complications were observed due to the thinning of the posterior wall of the auditory canal.

Our audiological results are in line with previous studies [4, 13, 14, 23, 24, 26, 27, 30, 35, 36]. The mean gain in speech understanding was very high compared to the results in other studies, which also used the German monosyllabic Freiburger test at 65 dB [23–25, 29, 35, 36]. Bone conduction thresholds remained mainly stable (shifted less than 10 dB), but dropped in one patient directly after implantation (Pat. 6). This patient had a petrosectomy and recovered spontaneously during follow-up time. Presumably, Pat.6 had a noise effect on the inner ear due to extensive drilling of about four hours intraoperatively. One patient (Pat. 10) experienced a spontaneous BC-drop during follow-up, 84 months after implantation.

### Strengths and limitations of the study

The evaluation of quality of life and subjective patient satisfaction not only plays a major role in terms of therapy objectives, but also provides conclusions on psychological, social and technical aspects and their potential for improvement. Therefore, the strengths of this study lie in the analysis and evaluation of the QoL-data, especially from long-term users. We could demonstrate that–even up to 7 years after implantation–user satisfaction and quality of life were improved. Furthermore, the APSQ questionnaire was the first to be applied to a cohort of transcutaneous BCD long-term users. Data about daily wearing time of bone conduction devices is sparse. In this study, we determined a potential effect of different demographic parameters on hours of daily use.

Nonetheless, limitations of this retrospective study need to be emphasized in regard to the small sample size meeting our inclusion criteria and the time point of quality of life evaluation, which was taken at only one time point. For this reason, a quality of life survey in a larger patient cohort would need to be performed at different time points over a longer period of time in order to detect possible fluctuation in patient satisfaction during follow-up.

## Conclusion

Subjective benefit in hearing related QoL improved significantly after implantation of this transcutaneous BCD. Additionally, both satisfaction with the audio processor and wearing time were rated very high. In combination with a low rate of manageable minor adverse events and significantly improved audiological outcomes, the implant can be considered as a comfortable, safe and effective long-term option in hearing rehabilitation for patients suffering from CHL or MHL.

## Supporting information

S1 Dataset Input data. (XLSX)

S1 Fig Unaided and aided scattergrams relating air-conductive pure-tone average to word recognition score. Scattergram relating unaided (A) and aided (B) air-conductive pure-tone average (PTA_4AC_) to word recognition score (WRS65) as recommended by the Hearing Committee of the American Academy of Otolaryngology–Head and Neck Surgery. PTA_4AC_ was plotted on the y-axis increasing 10 dB intervals from 0 to 110 dB HL (audiometer threshold). WRS65 was plotted on the x-axis increasing 10% in descending order from 100 to 0%.
(JPG)
