# Supplementary figures and images for: Patient-reported long-term benefit with an active transcutaneous bone-conduction device
Source: PLoS One. 2020 Nov 2;15(11):e0241247. doi: 10.1371/journal.pone.0241247 (PMC7605656; doi:10.1371/journal.pone.0241247)

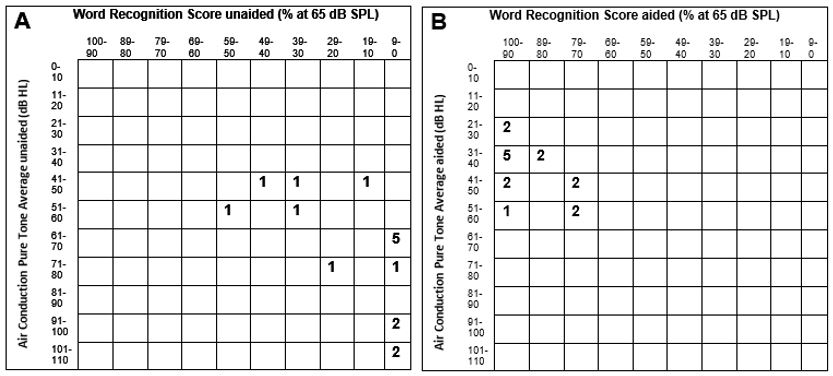

Supplement: S1 Fig — Scattergram relating unaided (A) and aided (B) air-conductive pure-tone average (PTA4AC) to word recognition score (WRS65) as recommended by the Hearing Committee of the American Academy of Otolaryngology–Head and Neck Surgery. PTA4AC was plotted on the y-axis increasing 10 dB intervals from 0 to 110 dB HL (audiometer threshold). WRS65 was plotted on the x-axis increasing 10% in descending order from 100 to 0%. (JPG) [file pone.0241247.s002.JPG]
